# Daily Protein Restriction Under Different Protein Levels in Whiteleg Shrimp (*Penaeus vannamei*): Growth, Digestive System, Gene Expression, and Health

**DOI:** 10.1155/anu/5126953

**Published:** 2026-07-29

**Authors:** Ebrahim Sotoudeh, Leila Khalili, Maryam Zoab, Zahra Bahmani, Noah Esmaeili

**Affiliations:** ^1^ Department of Fisheries, Faculty of Nano and Bio Science and Technology, Persian Gulf University, Bushehr 75169, Iran, pgu.ac.ir; ^2^ Anti-Aging and Regenerative Medicine Research Institution, School of Life Sciences and Medicine, Shandong University of Technology, Zibo 255049, China, sdut.edu.cn

**Keywords:** antioxidants, aquaculture, crustacean, energy turnover, health status, protein homeostasis, protein nutrition

## Abstract

Daily protein restriction, for the first time, under various protein levels for 8 weeks, was tested on growth, hemolymph parameters, digestive and serological enzyme activities, antioxidant defense, immune system, and its relative genes in whiteleg shrimp (*Penaeus vannamei*) (initial weight: 1.73 ± 0.17 g). We hypothesized that the protein levels in a protein restriction schedule can be decreased to maximize the benefits of protein restriction. The results indicate that a schedule of feeding 1 day of dietary 390 g/kg followed by 2 days of dietary 330 g/kg (1P392P33) is possible without significant impairment of growth, feed efficiency, digestive enzymes, immune system, and antioxidant defense. Further, the P26, P33, and 1P392P26 groups showed a decline (*p* < 0.05) in growth, flesh quality, and health, showing that dietary 260 and 330 g/kg protein is not enough for this species, even when we put this diet in a protein restriction schedule. Any whiteleg shrimp group fed dietary 260 g/kg protein experienced lower protease activity (*p* < 0.05), highlighting the impact of protein malnutrition on this digestive enzyme. These groups also had lower catalase activity and expression of *penaeidin-3a* and *lysozyme* genes (*p* < 0.05). There was no clear trend in hemolymph parameters among groups, except for lower triglycerides in experimental groups (but not 1P392P33) than in the control. In conclusion, feeding 66% of the farming period of whiteleg shrimp with the dietary 330 g/kg protein and the rest of the time with the dietary 390 g/kg protein is possible without growth, flesh quality, and health impairment.

## 1. Introduction

Whiteleg shrimp (*Penaeus vannamei*) was the most produced species in aquaculture worldwide in 2022 [1]. The farming system of this species after the postlarvae is totally dependent on artificial feed [1]. Considering the feed conversion ratio (FCR) of 1.5, more than 6.8 million tons of production for this species in 2022 [1], and a survival rate of 90%, around 11.3 million tons of feed are consumed to farm whiteleg shrimp each year. The requirement of protein for this species is around 400 g/kg [2, 3], which means that 4.5 million tons of protein are used each year for whiteleg shrimp production. Protein is the most expensive nutrient in whiteleg shrimp diets and should be supplied at optimal quality and quantity to provide economic benefits as well as growth, water quality, and health [1]. When diets contain excessive protein, resources are wasted, and environmental issues (such as ammonia toxicity) arise. However, “sub‐optimal” dietary protein levels can impair whiteleg shrimp growth, feed efficiency, and well‐being [4, 5];. Therefore, any effort to reduce protein usage in the feed of whiteleg shrimp, even a 5%–10% reduction in protein usage without negative impacts on shrimp growth and health, is a great achievement. Protein restriction is one of the most common ways to do so. Previous studies in crustaceans and fish showed that if this feeding strategy is done at the right time and manner, it can reduce protein usage substantially [6]. The main obstacle is that the right protein level and schedule should be applied, which sometimes results in maximum growth, but immunity and health can be impaired. Growth, feed intake, and feed efficiency did not decrease when Chinese shrimp (*Fenneropenaeus chinensis*) were fed 300 g/kg protein for 2 weeks and then 450 g/kg protein for 4 weeks [7]. There was no decrease in weight gain, feed efficiency, digestive enzyme activities, or crude protein content in whiteleg shrimp under 3 out of 6 weeks of a protein‐restricted diet plan (360 vs. control 430 g/kg) [8]. Feeding half of the farming period with protein restriction was only applicable in one of these experiments, and these investigations were not lengthy enough. We discovered that whiteleg shrimp should be fed a diet with 400 g/kg protein for at least 6 out of the 8 weeks if fasting is also applied (the remaining 2 weeks were 1 week of fasting and 1 week of feeding with a diet of 350 g/kg protein) [9]. A definitive conclusion could not be drawn from the conducted studies due to the short study period and the inconsistent outcomes.

To the best of our knowledge, no study has tested the impacts of daily protein restriction in aquaculture species, and the previous studies have been in weekly schedules [7–9]. Further, the impacts of selecting different protein levels in protein restriction are unknown and are investigated for the first time in the current study. In the present research, we tested the two different protein levels (330 and 260 g/kg), plus a control (390 g/kg), under daily protein restriction schedules in whiteleg shrimp. Thus, the purpose of this study was to examine the effects of varying protein levels and daily protein restriction on whiteleg shrimp growth performance, feed efficiency, survival rate, body composition, hemolymph parameters, immune system, antioxidant activity, and immune system parameters and their relative gene expressions. Another aim is to find the lowest possible protein levels that can be used in protein restriction to reduce protein usage during the rearing period.

## 2. Materials and Methods

### 2.1. Ethics Statement

To reduce the stress experienced during the handling of the whiteleg shrimp, all experimental procedures were closely adhered to the established guidelines outlined in the Declaration of Helsinki (1975), the Society for Animal Care and Use Guidelines (1998), and the national ethical framework for animal research in Iran [10]. However, it should be mentioned that animal ethics for crustaceans in several countries are not required so far.

### 2.2. Shrimp, Farming, and Experimental Design

The study was carried out in Bushehr, Iran, at the Persian Gulf University’s Laboratory of Aquatic Research. For a period of 14 days, the shrimp were fed a starting diet (pellet size: 0.8–1.4 mm; protein: 390 g/kg, fat: 120 g/kg, and ash: 90 g/kg) provided by the Behsan Taghzieh Arian Company (BTA Group). The shrimp was supplied by a local farm company (Boston Khalij, Bandar‐e Deylam, Iran). Prior to the experiment, whiteleg shrimp were visually inspected to ensure they were disease‐free, and only healthy individuals were chosen. During the intermolt stage, shrimps were moved to experimental fiberglass tanks (300 L, 90 cm diameter, 200 L water capacity) in accordance with the previous protocol [11]. A total of 360 shrimp (initial weight: 1.73 ± 0.17 g) were divided among 18 tanks, each containing 20 shrimp. Approximately 15% of the water volume was replaced daily in the tanks with filtered and disinfected saltwater (salinity: 36 ± 0.8 ppt). Water quality metrics were monitored and maintained at standard levels of 0.08 ppm for total ammonia nitrogen (Desun Uniwill, China), 7.6 ± 0.5 for pH (Hanna, HI 98128, USA), and 28.0 ± 0.8°C for temperature. The shrimp were given the experimental diets at a satiation level three times a day (09:00, 13:00, and 18:00) in natural light for the duration of the 8‐week trial. One of the main obstacles to success in protein restriction usage is the reduction in the palatability of diets with low protein levels. A fixed feeding rate cannot cover this point, and because of that, the apparent satiation level was used in order to monitor this suppressed appetite. The experimental groups were: control group (fed dietary 390 g/kg protein for 8 weeks), P33 (fed dietary 330 g/kg protein for 8 weeks), 1P392P33 (fed with dietary 390 g/kg protein for 1 day followed by 2 days dietary 330 g/kg protein), P26 (fed dietary 260 g/kg protein for 8 weeks), 1P392P26 (fed with dietary 390 g/kg protein for 1 day followed by 2 days dietary 260 g/kg protein), and P39‐P33‐26 (fed with dietary 390 g/kg protein for 1 day followed by 2 days dietary 330 g/kg protein, and then 1 day fed with dietary 390 g/kg protein followed by 2 days dietary 260 g/kg protein) (Table 1). The schedule of 1 day of feeding with optimum protein and 2 days of feeding with suboptimum protein was selected as the main feeding schedule, as this scheme worked well in whiteleg shrimp (unpublished data) and was used in another study as well [12].

**Table 1:** Experimental design for protein restriction with different protein levels in whiteleg shrimp was fed for 8 weeks.

Treatments	Day 1 (%)	Day 2 (%)	Day 3 (%)	Day 4 (%)	Day 5 (%)	Day 6 (%)	Day 7 (%)
Control	P39	P39	P39	P39	P39	P39	P39
33% Protein	P33	P33	P33	P33	P33	P33	P33
1P392P33	P39	P33	P33	P39	P33	P33	P39
26% Protein	P26	P26	P26	P26	P26	P26	P26
1P392P26	P39	P26	P26	P39	P26	P26	P39
P39‐P33‐26	P39	P33	P33	P39	P26	P26	P39

*Note:* P39%, protein 39%; P33%, protein 33%; P26%, protein 26%

### 2.3. Experimental Diets

To investigate protein restriction, three experimental diets with 390, 330, and 260 g/kg protein were formulated for this investigation (Table 2). Investigations have shown that 380–400 g/kg protein is optimum for whiteleg shrimp [2]. The lower levels of protein were also selected based on previous studies [2] and our pretrial test. We provided the ingredients from local commercial marketplaces, which included fish meal, shrimp meal, squid meal, soybean meal, wheat flour, gluten, fish oil, and lecithin. The feed preparation method involved drying, mixing, and pelleting the nutritional components, which followed previous protocols [13]. A meat grinder (Electrokar EC‐1, Tehran, Iran) was used to create pellets that were the right size for the experimental shrimp. To reduce the moisture content to less than 10%, the pellets were spread out on trays and dried in an oven at 45°C for 24–48 h. The pellets were dried and then stored at 4°C in nylon bags until they were required for analysis and shrimp feeding. The experimental diets and their chemical compositions are displayed in Table 2.

**Table 2:** Experimental diets (g/kg) were used to feed whiteleg shrimp (*Penaeus vannamei*) under protein restriction with different protein levels.

Ingredients	39% Protein	33% Protein	26% Protein
Wheat, red W.	290	395	487.8
Soybean meal—48%	264.6	221.3	200
Gluten meal	100	50	0
Fish meal	243.4	230.2	211
Squid meal	12.9	14.4	12.2
Fish oil	26	26	26
Soybean oil	15	15	15
Safflower oil	5	5	5
Other ingredients^a^	63	63	63
Proximate analysis (g/kg)			
Crude protein	390.2	331.2	268.0
Crude lipid	90.0	8.95	8.74
Ash	115.1	100.2	98.9
Moisture	89.4	88.6	90.2
NFE	295.6	371.3	436.5
Fiber	20.2	19.2	19.0
Gross energy (kJ/g)^b^	18.18	18.07	17.61

*Note:* All the ingredients were supplied by Taam Sazan Co (Bushehr, Iran).

^a^Other ingredients (g/kg): lecithin: 10, shrimp meal: 10, DL‐methionine: 1, L‐lysine HCl: 1, choline chloride: 1, vitamin premix: 10, and mineral premix: 10. Mineral premix mg/kg of premix: magnesium, 6400; copper, 2000; ferrous, 11,000; zinc, 7000; selenium, 100; iodine, 300; cobalt, 50; natrium, 5000. ATA Company, Tabriz, Iran. Vitamin premix (IU/kg of premix): ascorbic acid, 350,000; retinol, 1,000,000,000; cholecalciferol, 500,000,000; tocopherols, 500,000; vitamin K3, 960,000; thiamine, 980,000; riboflavin, 800,000; pyridoxine, 990,000; folic acid, 950,000; cobalamin, 10,000; biotin, 20,000; niacin, 995,000; pantothenic acid, 980,000. The chemical composition of the used ingredients in this study was reported earlier [12].

^b^Gross energy values calculated based on 23.6 kj g^−1^ proteins, 39.5 kj g^−1^ lipid, and 17.2 kj g^−1^ carbohydrates [10]. Nitrogen‐free extract NFE was calculated as NFE = 100 – (crude protein + lipid + crude fiber + ash + moisture), on a fed basis.

### 2.4. Growth Performance and Biochemical Composition Assessment

All shrimp were fasted for a full day after the experiment. Cold ice water was used to anesthetize the animals. Final weight (g), weight gain (g), specific growth rate (SGR), FCR, daily feed intake (% body weight/day), protein efficiency ratio (PER), lipid efficiency ratio (LER), productive protein value (PPV) (%), and survival rate (%) were among the growth and feed performance indices that were measured. Nine shrimp per treatment (three shrimp per tank) were selected and kept at −20°C for additional examination of their biochemical composition. During the time of the lab experiment, two samples from each tank were pooled to reduce the impact of individual sampling (two samples were collected from each tank, and one sample from each tank was kept as a backup). Three made samples, which represented three tanks from each treatment, were used for further steps. Using AOAC standard procedures, the proximate composition of the diets and shrimp was measured [14]. Weight loss was used to calculate dry matter after homogenized samples were dried for 24 h at 105°C in an oven (AMB50; ADAM, Milton Keynes, UK). Crude protein (N × 6.25) was quantified using the Kjeldahl technique with an automated Kjeldahl system (BÜCHI, Auto‐Kjeldahl K‐370; Switzerland). Crude lipid was measured by extraction of ether using a Soxhlet (Barnstead/Electrothermal, UK), and the ash content was determined after incineration in a muffle furnace (Finetech, Shin Saeng Scientific, Paju‐si, Gyeonggi‐do, South Korea) at 550°C for 6 h. The growth performance formulas are as follows:

Specific growth rate SGR%  BW/day=Ln final weight– Ln initial weight/ days×100,

Weight gain g=Final body weight– initial body weight,

Feed efficiency: Weight gain g/feed intake g,

Daily feed intake DFI %BW/day= Total feed fed g×100/average body weight g×days,

PER: Protein efficiency ratio= Shrimp wet weight gaing/ protein intakeg crude protein,

LER: Lipid efficiency ratio= Shrimp wet weight gain g/ lipid intake g crude lipid,

PPV%: Productive protein value=Shrimp protein gain g crude protein/protein intake g crude protein×100,

Survival rate %=Final number of shrimps survived/initial number of shrimps stocked×100.

### 2.5. Digestive Enzyme Activities

Nine shrimp (per treatment) had their intestines (midgut) removed in order to measure the activity of intestinal digestive enzymes (these shrimp were used for hemolymph biochemistry, immune system components, antioxidant parameters, and serological enzymes as well). During the time of the lab experiment, two samples from each tank were pooled to reduce the impact of individual sampling (one sample from each tank was kept as a backup sample). Three made samples, which represented three tanks from each treatment, were used for further steps. Supernatants from thawed samples homogenized in 30 volumes (v/w) of cold buffer (50 mM mannitol, 2 mM Tris–HCl, pH 7.0) and centrifuged at 3300 × *g* for 3 min were used to measure the digestive enzyme activity. One unit of activity was equivalent to the release of 1 μmol of p‐nitrophenol per minute at 405 nm when lipase activity was measured using p‐nitrophenyl myristate as a substrate [15]. Bernfeld’s [16] method, which used starch as the substrate, was used to measure amylase activity. The amount of enzyme needed to release 1 µmol of maltose in a minute was considered a single unit of amylase activity. Specific activity (*U*) was expressed as amylase activity = (maltose released [µmol])/(3 × mg protein) [17]. Total protease activity was assayed using casein as the reaction substrate, following the method described earlier [18]. The protein content of the examined samples was measured using the Bradford technique [19].

### 2.6. Hemolymph and Hepatopancreas Sample Preparation

To examine hemolymph biochemistry, immune system components, antioxidant parameters, and serological enzymes, nine shrimp were sampled from each tank (those were used for digestive enzyme activity as well). Hemolymph was extracted directly from the heart sinus using sterile syringes and immediately transferred to cooled centrifugal tubes. After being cooled at 4°C for 4 h to encourage coagulation, the hemolymph tubes were centrifuged at 2500 × *g* for 10 min at 4°C to obtain the serum. The hemolymph supernatant (serum) was then collected for additional examination at −80°C for less than 3 months [11]. At the same time, the 9 hepatopancreas were quickly removed, rinsed in ice‐cold phosphate buffer (100 mM potassium phosphate, pH 7.4; 100 mM potassium chloride; 1 mM ethylenediaminetetraacetic acid [EDTA]), and cryopreserved in liquid nitrogen before being stored at −80°C. During the time of the lab experiment, two samples from each tank were pooled to reduce the impact of individual sampling (one sample from each tank was kept as a backup sample). Three made samples, which represented three tanks from each treatment, were used for further steps.

### 2.7. Antioxidant Enzyme, Hemolymph Biochemistry, and Immune Parameters

Hepatopancreatic antioxidant enzyme activities were determined following a protocol established in the previous work [20]. In short, frozen hepatopancreas samples were homogenized for 30–45 s using a homogenizer in ice‐cold 100 mM phosphate buffer (9 mL buffer per gram of tissue). After centrifuging the homogenates at 12,000 × *g* for 30 min at 4°C, the supernatants were collected and kept at −80°C for further analysis, which was completed in less than 3 months [21]. Lipid peroxidation levels were measured using a modified thiobarbituric acid test. Trichloroacetic acid, butylated hydroxytoluene, and hydrochloric acid were used to react with the samples. After 15 min of heating to 100°C, they were centrifuged at 1000 × *g* for 10 min at room temperature. Using spectrophotometry at 535 nm, the amount of malondialdehyde (MDA) in the resulting supernatant was then determined by comparing it to a reference curve [22]. After adding the supernatant to a potassium phosphate‐based test combination containing hydrogen peroxide (H_2_O_2_), catalase activity was assessed by tracking the breakdown of H_2_O_2_ at 240 nm during a 3‐min period [23]. In particular, 1.9 mL of 0.05 M potassium phosphate and 1 mL of 0.059 M H_2_O_2_ (pH 7) were mixed with 100 μL of the supernatant, and the absorbance was measured. Using the Bradford method and bovine serum albumin as the standard, the soluble protein of the extract was measured (Bradford, 1976). Superoxide dismutase (SOD) activity was evaluated following the protocol described by [24]. For this assay, 500 μL of homogenate or serum samples were mixed with a reaction solution composed of 1300 μL carbonate buffer (pH 10.2), 500 μL nitro blue tetrazolium (60 μM), 100 μL Triton X‐100 (0.6%), and 100 μL hydroxylamine hydrochloride (20 mM, pH 6.0). The absorbance of the resulting solution was measured at 540 nm for 5 min at room temperature [24]. Hemolymph and hepatopancreas samples were analyzed for total protein, triglyceride, cholesterol, glucose, alkaline phosphatase (ALP) activity, aspartate aminotransferase (AST), alanine aminotransferase (ALT), lactate dehydrogenase (LDH), glutathione peroxidase (GPx), phenoloxidase, and acid phosphatase (ACP) using diagnostic kits from Nanjing Jiancheng Bioengineering Institute (Nanjing, China) in conjunction with an automatic biochemistry auto‐analyzer (Technicon RA‐1000, USA).

### 2.8. Relative Gene Expression Analysis

To evaluate the relative expression levels of *heat shock protein* (*HSP60*), *prophenoloxidase* (*ProPo*), *lysozyme*, *penaeidin-3a*, *lipopolysaccharide and β-1*,*3-glucan-binding protein* (*LGBP*), and *beta-actin* genes in the hepatopancreas (Table 3), nine frozen tissue samples per group were homogenized in lysis buffer. During the time of the lab experiment, two samples from each tank were pooled to reduce the impact of individual sampling (two samples were collected from each tank, and one sample from each tank was kept as a backup sample). Three made samples, which represented three tanks from each treatment, were used for further steps. Total ribonucleic acid (RNA) was extracted using a High Purity RNA Isolation Kit (Roche, Germany), and its quantity and quality were assessed via 1% agarose gel electrophoresis and a Nano Drop ND‐1000 spectrophotometer (Nano‐Drop Technologies, Wilmington, DE, USA). The selected RNA samples exhibited absorbance ratios (260/280 nm) between 1.86 and 2.00. Complementary DNA (cDNA) was synthesized from 1 µg of purified RNA using a 2‐step cDNA reverse transcription kit (CinnaGen, Iran) and a random hexamer primer. Gene‐specific primers were designed using the Primer3 online tool based on the nucleotide sequences of the target genes, with *beta-actin* serving as the housekeeping gene [20]. Quantitative real‐time PCR (RT‐PCR) was performed using a quantitative thermal cycler (Rotorgen2000, Corbett Research Australia) and RealQ Plus 2x Master Mix Green (amplicon, Denmark), with each sample analyzed in triplicate. The RT‐PCR cycling conditions were: initial denaturation at 95°C for 15 min, followed by 40 cycles of denaturation at 95°C for 20 s, annealing at 58°C for 30 s, and extension at 72°C for 30 s. Melting curve analysis was conducted by gradually increasing the temperature from 55 to 95°C at a rate of 0.5°C per 30 s. The same thermal cycling parameters were used for all genes. The presence of single amplicons was confirmed by agarose gel electrophoresis of the final PCR products. PCR efficiencies were determined using a five‐point logarithmic serial dilution with three replicates. The relative abundance of mRNA transcripts was determined from 3 independent biological replicates, each run in triplicate to obtain threshold cycle (Ct) values [20]. Gene expression data were analyzed using the 2^−ΔΔCT^ method [25].

**Table 3:** Quantitative real‐time PCR primer sequences and amplification efficiencies for target genes in whiteleg shrimp.

Gene	Sequences of primers (5′ → 3′)	Gene bank accession number	PCR product size	Efficiency (%)
*Lysozyme*	F:TGT TCC GAT CTG ATG TCC R:GCT GTT GTA AGC CAC CC	XM_070138434	123	98
*Beta-actin*	F:CCACGAGACCACCTACAAC R:AGCGAGGGCAGTGATTTC	AF300705.2	142	97
*Penaeidin-3a*	F:CACCCTTCGTGAGACCTTTG R:AATATCCCTTTCCCACGTGAC	XM_027360479	141	96
*HSP60*	F: CAGACTCCATGCCACACCAT R: CTGTGCGAACAACCTTGGTG	XM_070123420	166	90
*Prophenoloxidase*	F:GAGATCGCAAGGGAGAACTG R:CGTCAGTGAAGTCGAGACCA	XM_027379986	141	96
*LGBP*	F: CAGGGGCAACGACAACTTTG R: TGAGTACTCGACGTGGGTCT	XM_070119658	112	92

Abbreviation: *LGBP*, *lipopolysaccharide and β-1,3-glucan-binding protein*.

### 2.9. Statistical Analysis

Different treatment groups, each with three replicates, were used in this fully randomized design investigation. Before the analysis, Shapiro–Wilk and Levene’s tests were used to confirm the homogeneity of variance and the normality of the data distribution, respectively. One‐way ANOVA in SPSS (Version 22.0 for Windows) was then used to examine the data. Growth performance, body composition, hemolymph parameters, digestive enzymes, immune system responses, antioxidant activities, serological enzyme levels, and gene expression were compared among the treatment groups using a Tukey’s Honest Significant Difference (HSD) post hoc test when ANOVA revealed significant differences at the level of 0.05 [26]. No outlier was detected in our data based on the *Z*‐score method.

## 3. Results

### 3.1. Growth Performance and Body Composition

The growth performance data for whiteleg shrimp fed daily schedules of dietary protein restriction under different protein levels are shown in Table 4. There was no significant difference in FCR, LER, PPV (%), and survival rate among groups. The control and 1P392P33 groups did not differ in final weight (g), weight gain (g), SGR, and feed intake (g). Other treatments were significantly lower than these two groups in the mentioned parameters. Also, PER in the P26 treatment had higher values compared to other groups (*p* < 0.05) (Table 4).

**Table 4:** Growth performance of whiteleg shrimp (*Penaeus vannamei*) experienced protein restriction under various protein levels for 8 weeks.

Parameters	Control	P33	1P392P33	P26	1P392P26	P39‐P33‐26
Final weight (g)	11.20 ± 1.08^a^	8.95 ± 0.55^c^	10.64 ± 1.03^ab^	8.66 ± 0.65^c^	9.50 ± 0.95^bc^	9.35 ± 1.05^bc^
Weight gain (g)	9.82 ± 0.63^a^	7.15 ± 0.71^c^	9.18 ± 0.89^ab^	6.90 ± 0.70^c^	7.87 ± 0.91^bc^	7.84 ± 0.62^bc^
SGR (% BW/day)	3.42 ± 0.12^a^	2.83 ± 027^b^	3.46 ± 0.13^a^	2.81 ± 0.21^b^	3.00 ± 0.38^b^	2.94 ± 0.10^b^
FCR	1.30 ± 0.06	1.51 ± 0.14	1.37 ± 0.11	1.52 ± 0.14	1.45 ± 0.12	1.46 ± 0.10
Feed intake	12.71 ± 0.36^a^	10.75 ± 0.67^b^	12.53 ± 0.20^a^	10.40 ± 0.21^b^	11.38 ± 0.66^ab^	11.48 ± 1.58^ab^
PER	2.21 ± 0.26^c^	2.53 ± 0.18^bc^	2.39 ± 0.20^bc^	3.20 ± 0.21^a^	2.70 ± 0.21^b^	2.47 ± 0.09^bc^
LER	9.81 ± 1.16	9.27 ± 0.67	9.43 ± 0.77	9.24 ± 0.61	9.29 ± 0.73	9.07 ± 0.32
PPV (%)	36.69 ± 0.37	32.44 ± 0.62	35.32 ± 5.61	39.99 ± 6.04	35.07 ± 5.25	34.49 ± 0.97
Survival rate (%)	98.41 ± 2.75	98.41 ± 2.75	100.00 ± 0.0	96.83 ± 2.75	98.41 ± 2.75	100.00 ± 0.0

*Note:* Values are represented by means ± SDM of triplicate tanks; means without letter labels are not significantly different. According to the Tukey range test, the letters a, b and c indicate significant differences in the treatments (*p* < 0.05).

Abbreviations: FCR, feed conversion ratio; LER, lipid efficiency ratio; PER, protein efficiency ratio; PPV, productive protein value; SGR, specific growth rate.

The ash and moisture contents of the shrimp body were not changed significantly (Table 5). However, a clear trend was observed in the protein level within the body composition of the shrimp. Specifically, the control had a higher level of protein than the P33, P26, and 1P392P26 treatments (*p* < 0.05). Lipid levels in the body of the P26 shrimp were increased compared to other treatments (Table 5).

**Table 5:** The proximate composition (g/kg) of whiteleg shrimp (*Penaeus vannamei*) experienced protein restriction under various protein levels for 8 weeks.

Proximate analysis	Control	P33	1P392P33	P26	1P392P26	P39‐P33‐26
Crude protein	141.6 ± 7.9^a^	123.5 ± 7.4^b^	128.5 ± 7.8^ab^	119.8 ± 9.4^b^	119.6 ± 7.2^b^	128.4 ± 8.1^ab^
Crude lipid	32.5 ± 3.2^b^	36.7 ± 2.1^b^	32.6 ± 5.0^b^	49.4 ± 4.6^a^	37.5 ± 2.1^b^	34.5 ± 1.8^b^
Ash	33.9 ± 2.7	31.0 ± 3.3	32.5 ± 2.7	29.8 ± 3.1	31.1 ± 3.0	31.3 ± 1.8
Moisture	743.9 ± 14.8	765.1 ± 14.4	753.6 ± 13.8	766.2 ± 9.3	763.4 ± 6.8	772.6 ± 13.7

*Note:* Values are represented by means ± SDM of triplicate tanks; means without letter labels are not significantly different. According to the Tukey range test, the letters a and b indicate significant differences in the treatments (*p* < 0.05).

### 3.2. Digestive Enzymes

**Figure 1:**
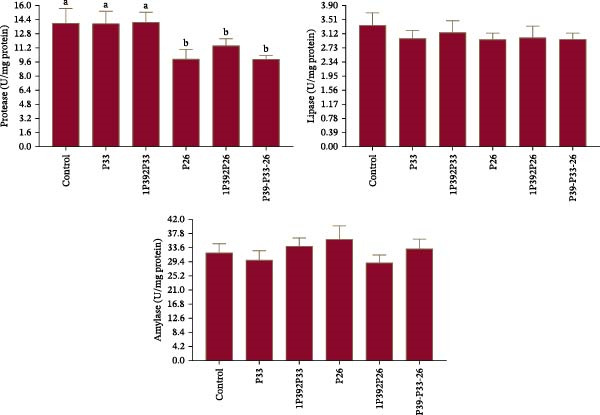
Digestive enzyme activity of whiteleg shrimp experienced protein restriction under various protein levels. Significant differences in treatment are indicated by letters a, b, and c based on the Tukey range test (*p* < 0.05). Values are represented by means ± SDM of three tanks.

The digestive enzyme activity in whiteleg shrimp under daily protein limitation at various protein levels is shown in Figure 1. Protease activity (U/mg protein) in the control, P33, and 1P392P33 groups was higher than in other treatments (*p* < 0.05). No significant changes in lipase and amylase activities among shrimps were observed.

### 3.3. Chemical Parameters of the Hemolymph

**Figure 2:**
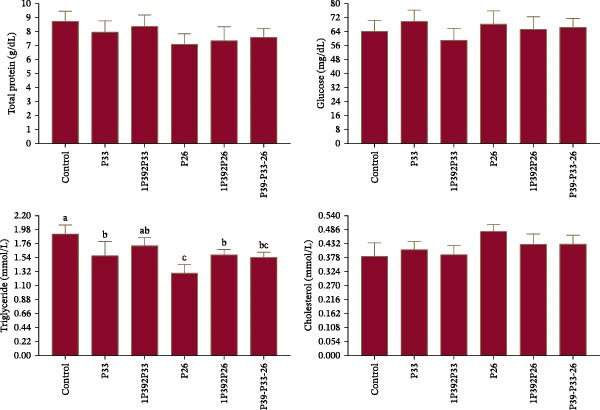
Hemolymph chemical parameters of whiteleg shrimp experienced protein restriction under various protein levels. Significant differences in treatment are indicated by letters a, b, and c based on the Tukey range test (*p* < 0.05). Values are represented by means ± SDM of three tanks.

Figure 2 illustrates the observed changes in total protein, triglyceride, cholesterol, and glucose levels across the various scheduled daily protein restrictions under different protein levels. The current data did not indicate any significant differences in total protein, cholesterol, and glucose among the treatments. However, triglyceride levels in the control and 1P392P33 groups were elevated compared to the P26 treatment (*p* < 0.05).

### 3.4. Immune System Parameters and Relative Gene Expressions

**Figure 3:**
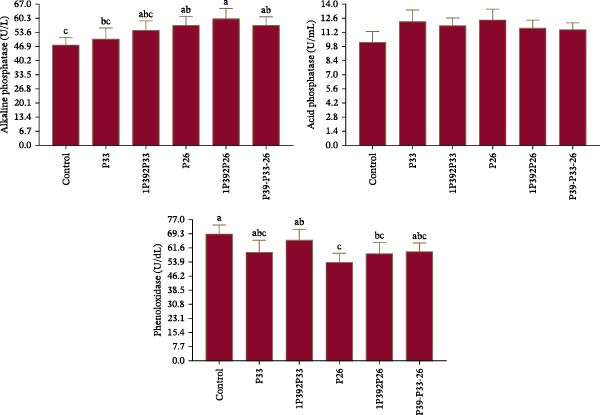
Immune system parameters of whiteleg shrimp experienced protein restriction under various protein levels. Significant differences in treatment are indicated by letters a, b, and c based on the Tukey range test (*p* < 0.05). Values are represented by means ± SDM of three tanks.

The results for immune system‐related factors are shown in Figure 3. The data demonstrated that while ACP (U/mL) was not changed by protein restriction or quantity, ALP (U/L) in the control and P33 groups was lower than the 1P392P26 treatment (*p* < 0.05). Phenoloxidase activity (U/dL) in whiteleg shrimp under the schedule of control and 1P392P33 feeding was higher than in the P26 group (*p* < 0.05).

**Figure 4:**
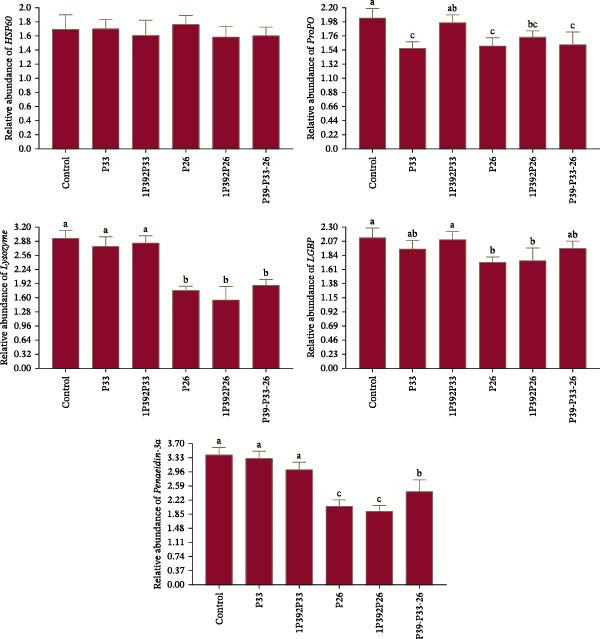
Genes related to the immune system of whiteleg shrimp experienced protein restriction under various protein levels. Significant differences in treatment are indicated by letters a, b, and c based on the Tukey range test (*p* < 0.05). Values are represented by means ± SDM of three tanks.

All measured genes, but not *HSP60*, were significantly altered by daily protein restriction under various protein levels (Figure 4). The expression of *ProPO* in the control and 1P392P33 groups experienced an increase compared to other treatments but not the 1P392P26 group. *LGBP* expression in those whiteleg shrimps was farmed under control, and 1P392P33 feeding schedules were higher than P26 and 1P392P26 groups (*p* < 0.05). *Lysozyme* and *penaeidin-3a* expression were similarly upregulated in the control, P33, and 1P392P33 groups compared to other treatments (*p* < 0.05).

### 3.5. Antioxidant Activities

**Figure 5:**
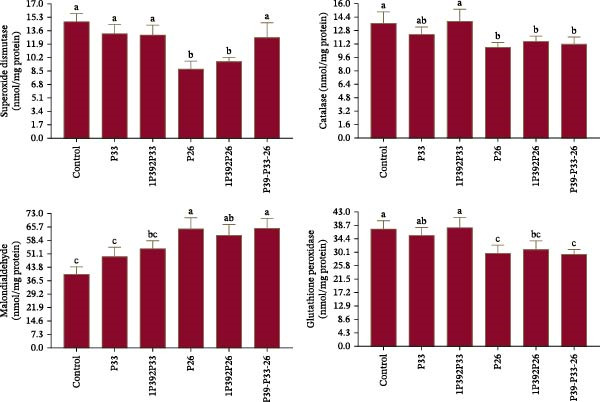
Antioxidant activities of whiteleg shrimp experienced protein restriction under various protein levels. Significant differences in treatment are indicated by letters a, b, and c based on the Tukey range test (*p* < 0.05). Values are represented by means ± SDM of three tanks.

The present research showed that the activity of all measured antioxidant parameters was changed (Figure 5). Catalase and GPx activities in the control and 1P392P33 groups were greater than those of other treatments but not P33 shrimps (*p* < 0.05). Control, P33, 1P392P33, and P39‐P33‐26 groups showed higher levels of SOD than others (*p* < 0.05). Finally, MDA was decreased in the control, P33, and 1P392P33 groups in comparison to the P26 and P39‐P33‐26 treatments (*p* < 0.05).

### 3.6. Liver Enzymes

**Figure 6:**
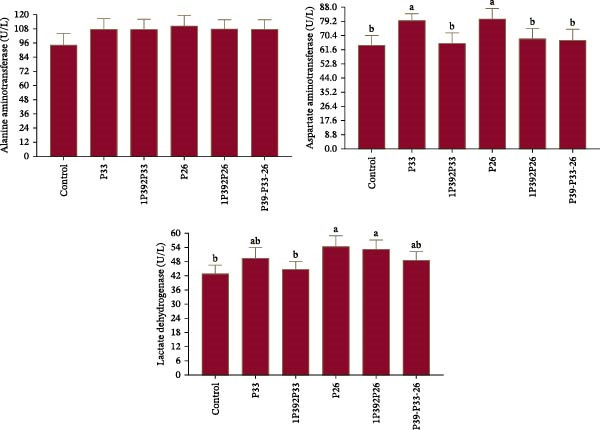
Serological enzyme parameters of whiteleg shrimp experienced protein restriction under various protein levels. Significant differences in treatment are indicated by letters a, b, and c based on the Tukey range test (*p* < 0.05). Values are represented by means ± SDM of three tanks.

Figure 6 shows the alterations in liver enzymes (ALT, AST, and LDH) in whiteleg shrimp that were subjected to varied daily schedules of limited protein. While ALT remained unchanged across experimental groups, AST and LDH were altered significantly. AST in the P33 and P26 groups demonstrated higher values compared to others (*p* < 0.05). LDH followed approximately the same trend, so that P26 and 1P392P26 treatments had higher levels than control and 1P392P33 groups (*p* < 0.05).

## 4. Discussion

Protein restriction may lower feed costs while simultaneously enhancing water quality without compromising the health and growth rates. The current research, as a follow‐up of our project related to protein restriction in whiteleg shrimp, tried to broaden the number of days feeding with restricted protein diets and manipulate protein levels to test whether we can lower protein levels in protein restriction schedules. The results indicated that applying 1 day of feeding with 390 g/kg, followed by 2 days of feeding with the dietary 330 g/kg (1P392P33) protein, without decreasing growth, flesh quality, and health, is possible. When the same schedules were conducted under 260 g/kg protein (1P392P26) or the combination of three protein levels (P39‐P33‐26), the growth, flesh quality, and health were impaired. Further, those fed a diet of 260 g/kg protein in the whole period of the experiment experienced growth reduction. Previous studies clearly reported the insufficiency of 260 g/kg protein in whiteleg shrimp [2, 3]. Based on previous trials (unpublished data) and the current study, we can claim that 330 g/kg is a threshold for this daily protein restriction schedule, and less than this, growth and health are compromised.

### 4.1. Growth Performance and Proximate Composition

Protein usage in aqua feeds can be decreased without compromising growth and feed efficiency when protein restriction is implemented properly at the right time and in the right manner. Protein restriction under weekly schedules has already been studied in crustaceans without growth reduction [7, 8], which could maximally reduce 7% protein usage (with 360 and 430 g/kg in a 6‐week farming period). Whiteleg shrimp needed to be fed at least 6 out of 8 weeks with a dietary 400 g/kg protein (the remaining 2 weeks were 1 week of fasting and 1 week of feeding with a dietary 350 g/kg) in order to provide insignificant growth compared to the control [9]. Based on Jahangiri et al. [9], it was not a beneficial idea to include 1 week of fasting during protein restriction. Also, testing daily protein restriction was suggested in that study. In the current research, we discovered that the daily restricted protein schedule can further reduce protein usage so that feeding with 390 g/kg protein for 1 day and 330 g/kg protein for 2 days was appropriate without having an adverse effect on the growth performance and feed efficiency.

Protein restriction might be applied to crustaceans without adversely influencing feed efficiency and growth, in line with our research. Chinese shrimp (1.3 g) were fed a diet containing 290 g/kg protein for 2 weeks, followed by a diet containing 440 g/kg protein for 4 weeks. In this particular experimental design, weight gain, feed efficiency, and PER did not decrease [7]. When compared to those fed control (430 g/kg protein), the growth performance of whiteleg shrimp under restricted dietary protein (360 g/kg) was unaffected for 3 of the 6 weeks [8]. Furthermore, crayfish (*Cherax cainii*) were fed a diet of 170 g/kg protein for 2 weeks and then a diet of 360 g/kg protein for 10 weeks. Their results demonstrated that protein restriction hindered growth and altered the composition of the gut microbiota [27]. These studies demonstrate that protein restriction is feasible in crustaceans, even if their experimental designs differ. As can be seen, a variety of weekly plans of protein restriction were examined, but none of them evaluated daily protein restriction schedules in fish or crustaceans, making it difficult to compare their findings with the available data. Weekly schedules of protein restriction are simpler to implement, which may be why earlier investigations examined them [8, 13, 28, 29]. If daily plans are set up rather than weekly ones, animals may not change their metabolisms during protein restriction. Further research is necessary; however, amino acid deprivation throughout weekly schedules may result in decreased protein synthesis and ultimately declined growth. Whiteleg shrimp are able to maintain proper homeostasis in daily schedules because they are regularly supplied with amino acids from the dietary 390 g/kg protein. Therefore, protein restriction should be carried out in the appropriate schedules and times. If the period of feeding with “not‐optimum protein levels” exceeded the animal’s threshold (3 out of 5 weeks, for example, has been for the majority of cases in prior experiments), growth and feed efficiency were decreased. Besides, the protein levels during protein restriction time also matter, as our study indicated that feeding a diet with 260 g/kg protein with the same schedules decreased growth and feed efficiency, but dietary 330 g/kg protein did not. Many more investigations are required in other species to find the best schedules, protein levels, and time period of protein restriction.

The muscle composition of crustaceans is affected by various factors, including age, size, gender, water quality, and environmental variables, while feed often exerts a more significant influence. The protein levels in the body were significantly altered, resulting in decreased protein concentrations for those fed a diet of 260 g/kg over 8 weeks. The current data and earlier investigations have revealed a detrimental aspect of protein restriction on the body proximate composition. The results of the present investigations align with prior studies [7, 9] and, in contrast to others [13, 27, 28]. Measuring proximate composition is recommended in protein restriction research, as it serves as a significant predictor of flesh quality and is likely to be changed under protein restriction. Consuming foods with high quantities of protein and omega‐3 fatty acids can enhance human health. In trials involving protein limitation, animals are susceptible to flesh quality impairment, a problem that needs consideration.

### 4.2. Digestive Enzymes

The activity of digestive enzymes serves as a reliable indicator of nutrient absorption in animals [30]. Hence, whiteleg shrimp exhibiting robust digestive enzyme profiles may demonstrate enhanced growth rates and improved feed efficiency. In the present study, protease activity correlated with dietary protein levels, revealing that shrimp consuming 260 g/kg protein had reduced protease levels compared to other groups. The growth and health of whiteleg shrimp in these groups were compromised, indicating significant difficulties with nutrient digestion and the application of these nutrients for maintaining homeostasis. On the other hand, digestive enzyme activities in the best treatment of our study (1P392P33) did not differ from the control. Research findings indicated that digestive enzyme activities and protein levels were unchanged when growth performance was unaffected under protein restriction treatment [8]. Furthermore, in their study, whiteleg shrimp subjected to severe protein restriction for 4 out of 6 weeks exhibited reduced growth; however, digestive enzyme activities remained unaffected, which contradicts our findings. These studies, in line with the current research, suggest that the relationship between growth and digestive enzymes is blurred, necessitating further investigation across many species. Animals in these studies consume at least 2 protein levels, which can directly influence digestive enzymes, especially protease, and this makes the understanding of nutrient utilization during protein restriction more complicated. An interesting line of research can be stimulating digestive enzyme activities in the protein restriction groups by some supplements, such as herbal medicine [31], citric acid [32], and probiotics [33]. Perhaps an increase of digestive enzymes, such as protease, by supplements can result in improved growth and health in the restricted protein group, eventually allowing us to rear shrimp for more days with dietary restricted protein.

### 4.3. Health Status

Hemolymph in crustaceans is a vital fluid that has the duty of transportation of nutrients among organs [34]. Hemolymph functions as blood for crustaceans, and evaluating biochemical markers within it might indicate the physiological and immunological status of these species [35]. In the current study, triglycerides were depleted in all treatments but not in the 1P392P33 group. Triglyceride is the major component in the hepatopancreas lipid. We can hypothesize that triglycerides in those fed a dietary 260 g/kg protein were used for the energy demands, but it is more likely that not enough energy was provided. When we match this data with health and growth performance (they were impaired in those fed a dietary 260 g/kg protein during the protein restriction period), this phenomenon makes more sense. Similar to the present investigation, depletion of triglycerides and cholesterol during weekly protein restriction in whiteleg shrimp occurred [9], which is in line with the current research. Whiteleg shrimp had a 70% feed limitation during the postlarval stage for 3 days, followed by cultivation with varying dietary protein levels (300, 370, and 430 g/kg) for a duration of 70 days [36]. Lage et al. [36] reported that triglyceride levels were lower in those consuming a diet comprising 370 and 300 g/kg protein, which is in line with the present study. Similarly, other researchers reported that when protein was not provided at an optimum level, triglyceride levels declined compared to the control. For example, in whiteleg shrimp [37], hybrid grouper (♀ *Epinephelus fuscoguttatus* × ♂ *Epinephelus lanceolatus*) [38], Holland carp (*Spinibarbus hollandi*) [39], blackspotted croaker (*Nibea diacanthus*) [40], and golden mandarin fish (*Siniperca scherzeri*) [41], the same results were observed. The possible mechanism can be a coordinated response that reduces very‐low‐density lipoprotein (VLDL) secretion and accelerates peripheral triglyceride clearance through increased hepatic expression of Apolipoprotein A5 (APOA5) via triggering the PERK‐CREBH‐APOA5 axis, increasing the activity of peripheral lipoprotein lipase, and reducing amino acid availability to produce triglycerides. This accelerated clearance reduces the overall levels of circulating triglycerides [42]. In the present data, the absence of alterations in hemolymph biochemistry parameters between the control and 1P392P33 groups indicates that energy reserves may not have needed activation. The lack of any difference in growth performance, feed intake, digestive enzyme, and health status occurred between control and 1P392P33 treatments as well, which can be further evidence of well‐balanced energy homeostasis. Energy turnover and reserves are important in protein restriction research, as energy resources must be supplied on days when animals are given suboptimal protein. Future research is advised to examine how protein restriction may influence these parameters and energy homeostasis, as the present study is still preliminary.

Crustaceans rely on the immune system and antioxidant factors to sustain overall health and homeostasis [43]. Due to the protein‐based molecular structure of critical immune factors and antioxidant components, it is recognized that a sufficient amount and optimal quality of proteins are necessary [44, 45]. This enables the assessment of crustacean health by monitoring parameters such as lysozymes, phenoloxidase, ACP, ALP, relative gene expressions, MDA, SOD, GPx, and catalase. In the present investigation, phenoloxidase activity in those shrimps under restricted protein schedules with a feeding of dietary 260 g/kg protein declined, but ALP in those groups was higher than in the control. These groups also experienced downregulation of *lysozyme*, *LGBP*, *penaeidin-3a*, and *ProPO* genes compared to the control. These changes show a sign of unhealthy conditions in whiteleg shrimp, which are matched with declined growth, digestive enzymes, and other health parameters in those fed a dietary of 260 g/kg protein. There is strong evidence for immune suppression based on both classic and molecular (gene expression) parameters. The immunosuppression has been a concern associated with protein restriction. Research on rainbow trout (*Oncorhynchus mykiss*) and Siberian sturgeon (*Acipenser baerii*) indicated that diminished immune parameters were associated with protein restriction [13, 28]. Moreover, in weekly protein restriction schedules for whiteleg shrimp, immune system suppression was seen [8, 9]. All these findings, along with the current data, suggest that protein restriction may lead to immune system suppression, especially when enough protein is not provided during restricted protein feeding. Other studies also likely reported that immune system suppression, along with decreased growth, occurs when aquatic species are not fed an optimum quantity of protein compared to the controls [41, 46–49]. While no change of immune response with feeding suboptimum protein was also observed [50, 51], most of the conducted studies showed that suppression of immune responses occurred. Testing immunostimulants to alleviate the side effects of protein restriction can be an interesting topic for future research.

The function of antioxidant activity is to neutralize unstable chemicals known as reactive oxygen species, which harm cells via oxidative stress. The present research, similar to the immune response, found that those groups that had dietary 260 g/kg in their protein restriction schedules possessed lower SOD, GPx, and catalase and higher MDA compared to the control and the best treatment (1P392P33). This is in line with other health status parameters and the growth performance of the current study. Suppression of antioxidant parameters and excessive lipid peroxidation are clearly signs of oxidative stress, and perhaps they have been the main reason for decreased growth. The body to tackle oxidative stress required energy, and those groups fed a dietary 260 g/kg during protein restriction already showed depletion of energy (shown by triglyceride). This means perhaps less energy was left toward balancing antioxidant defense in these treatments. Reduced SOD and GPx and higher MDA in our previous weekly protein restriction were observed [9], which is in line with the current data. Other studies also showed that feeding suboptimum protein levels that decreased growth also coincided with suppressed antioxidant defense. For example, in greasyback shrimp (*Metapenaeus ensis*) (340 vs. 390 g/kg protein) [52], whiteleg shrimp (300 vs. 340 g/kg protein) [53], (300 vs. 350 g/kg protein) [54], (340 vs. 490 g/kg protein) [50], and (320 vs. 400 g/kg protein) [47], redclaw crayfish (*Cherax quadricarinatus*) (280 vs. 350 g/kg protein) [55], and oriental river prawn (*Macrobrachium nipponense*) (380 vs. 420 g/kg protein) [56], the same results, similar to the current data, were reported. Conversely, no change in antioxidant defense in giant freshwater prawn [51], Nile tilapia [57], and yellow catfish (*Pelteobagrus fulvidraco*) [49] fed various protein levels was observed.

In summary, the aforementioned research and current data imply that protein restriction schedules and also feeding with suboptimum protein adversely affect immunity and antioxidant activity. A robust immune and antioxidant system is economically beneficial and can enhance survival rates, while animals with compromised immune and antioxidant systems are more susceptible to substantial mortality and financial losses under both acute and mild stressors.

Certain indicators, including ALT, AST, and LDH, are routinely assessed in crustacean research to evaluate the health status of the hepatopancreas. In the current investigation, LDH in those schedules with dietary 260 g/kg (P26, 1P392P26, and P39‐P33‐26) was lower than in the control. Accordingly, those shrimps fed a diet of 260 g/kg had higher AST and LDH compared to the control, which shows these groups suffer from impaired hepatopancreas homeostasis. However, there was no difference between the control and the best treatment of this study (1P392P33) in serological enzymes, which is in line with our previous study (the only available research on this topic so far) [9]. Other studies also showed that when aquatic species were fed suboptimum protein levels, some serological enzymes were elevated. For example, in whiteleg shrimp (320 vs. 400 g/kg protein) [47] and (300 vs. 340 g/kg protein) [53], the same findings were observed. In fish species such as gibel carp (*Carassius auratus* gibelio var) (290 vs. 370 g/kg protein) [58], blunt snout bream (*Megalobrama amblycephala*) (280 vs. 360 g/kg protein) [46], bighead carp (*Aristichthys nobilis*) (240 vs. 320 g/kg protein) [59], and golden mandarin fish (380 vs. 490 g/kg protein) [41], elevated serological enzymes with feeding suboptimum protein levels were reported. However, no change in serological enzymes in oriental river prawn when they were fed various protein levels was seen [56]. The key takeaway from these studies and our investigations is that serological enzymes can be raised when the protein level is not at the “optimum level.” The trend of changes of AST and LDH in the current data is matched with immune and antioxidant defense parameters. However, many more studies at the molecular and classic levels are required to illustrate how protein restriction can affect the health status.

## 5. Conclusions

After 8 weeks of farming whiteleg shrimp with daily protein restriction and under various protein levels, we can introduce 1 day of farming with protein 390 g/kg and 2 days with dietary 330 g/kg protein (1P392P33) as the best group, which was comparable to the group fed the whole period with dietary 390 g/kg protein. Applying the 1P392P33 schedule means feeding whiteleg shrimp with a dietary 330 g/kg for 66% of the farming period and the rest of the time with a dietary 390 g/kg without any problem in growth, flesh quality, and health. Accordingly, it means a 12% reduction in protein usage, which is a great number for the most‐produced aquaculture species. The results of the current study can have broader implications for aquaculture nutrition and feed management so that protein restriction can be applied more commonly in farms to benefit from reduced protein usage and economic gains. Investigations of daily schedules, coupled with the provision of additional energy, are advised in long‐term, field‐based studies. Molecular investigations, particularly omics, are necessary to identify the pathways and molecular activities implicated in protein restriction.

## Author Contributions

**Ebrahim Sotoudeh**: writing, resources, research, investigation, ideas, supervision, revision. **Leila Khalili**: writing, statistical analysis, research, investigation. **Maryam Zoab**: research, laboratory works, investigation. **Zahra Bahmani**: research, investigation. **Noah Esmaeili**: writing, validation, ideas, revision.

## Funding

There is no external funding to report.

## Ethics Statement

All experimental procedures involving animals in this study were conducted in strict accordance with the ethical regulations governing animal research within Iran [10]. Specifically, the methodology meticulously adhered to the comprehensive ethical principles stipulated in the Declaration of Helsinki (1975) and the detailed animal care and use guidelines established by the Society for Neuroscience (1998). These established regulations and guidelines ensure the ethical treatment and welfare of the animals involved in research.

## Conflicts of Interest

The authors declare no conflicts of interest.

## Data Availability

The data that support the findings of this study are available upon request from the corresponding author. The data are not publicly available due to privacy or ethical restrictions.
